# Physicochemical Properties and Antiherpetic Activity of κ-Carrageenan Complex with Chitosan

**DOI:** 10.3390/md21040238

**Published:** 2023-04-13

**Authors:** Viktoriya N. Davydova, Natalya V. Krylova, Olga V. Iunikhina, Aleksandra V. Volod’ko, Evgeniya A. Pimenova, Mikhail Y. Shchelkanov, Irina M. Yermak

**Affiliations:** 1 G.B. Elyakov Pacific Institute of Bioorganic Chemistry, Far-Eastern Branch of the Russian Academy of Sciences, 690022 Vladivostok, Russia; morskaia@list.ru (A.V.V.); imyer@mail.ru (I.M.Y.); 2 G.P. Somov Institute of Epidemiology and Microbiology, Rospotrebnadzor, 690087 Vladivostok, Russia; krylovanatalya@gmail.com (N.V.K.);; 3 A.V. Zhirmunsky National Scientific Center of Marine Biology, Far Eastern Branch, Russian Academy of Sciences, Palchevskogo 17, 690041 Vladivostok, Russia

**Keywords:** carrageenan, chitosan, polyelectrolyte complex, antiherpetic activity, selective index

## Abstract

Nanoparticles formation is one of the ways to modulate the physicochemical properties and enhance the activity of original polysaccharides. For this purpose, based on the polysaccharide of red algae, κ-carrageenan (κ-CRG), it polyelectrolyte complex (PEC), with chitosan, were obtained. The complex formation was confirmed by ultracentrifugation in a Percoll gradient, with dynamic light scattering. According to electron microscopy and DLS, PEC is dense spherical particles with sizes in the range of 150–250 nm. A decrease in the polydispersity of the initial CRG was detected after the PEC formation. Simultaneous exposure of Vero cells with the studied compounds and herpes simplex virus type 1 (HSV-1) showed that the PEC exhibited significant antiviral activity, effectively inhibiting the early stages of virus–cell interaction. A two-fold increase in the antiherpetic activity (selective index) of PEC compared to κ-CRG was shown, which may be due to a change in the physicochemical characteristics of κ-CRG in PEC.

## 1. Introduction

Polysaccharides of marine origin are macromolecules that have engaged researchers for many decades. Such attractiveness of these compounds is not accidental, since they combine biocompatibility and low toxicity with unique biological properties and low cost. In recent years, studies related to the development drugs for pharmaceuticals and biomedicine based on marine biopolymers have been carried out most intensively [1,2,3]. This is due to the activities found for them: antitumor [4], antibacterial [5], antiviral [6], anticoagulant [7] and a number of others [8].

Some of the well known polysaccharides of marine origin are the polysaccharides of red algae—carrageenans (CRGs). They are a family of sulfated copolymers of 3-linked β-D-galactopyranose and 4-linked α-D-galactopyranose (D-units) or 4-linked 3,6-anhydro-α-D-galactopyranose [9]. Depending on the number and position of sulfate groups, as well as the presence or absence of 3,6-anhydrogalactose in the molecule, CRGs are classified into various structural types, denoted by Greek letters. The most famous of which are κ-, λ- and ι-CRGs [10]. Carrageenans are the most studied among algae polysaccharides in terms of toxicity, pyrogenicity, and allergenicity [11]. Their safety of use for food and medical purposes has been confirmed by various studies of different scientific groups in experiments in vivo [12,13,14].

It is known that CRGs, mimicking heparan sulfate, are potential antivirals that can interfere with the early stages of viral replication, including virus entry, by masking the positive charge of the virus surface receptors to prevent them from binding to the heparan sulfate proteoglycans in the host cell surface [15]. CRGs can be included in pharmaceutical compositions for the prevention or treatment of viral infections with no side effects [16]. Earlier, we tested the effect of CRGs, isolated from a different family of red algae of the Pacific coast, at the stage of viral infection, and we showed the dependence of antiviral activity on the structural features of these polysaccharides [17]. The results of pre-treatment of herpes simplex virus type 1 (HSV-1) with CRGs and pre-treatment of cells with CRGs revealed that these polysaccharides affect the earliest stages of the viral life cycle, which are the attachment and the penetration ones. CRGs, containing 3,6-anhydrogalactose units, such as κ- and κ/β-CRGs, significantly reduced the attachment of HSV-1 to cells. However, they showed lower virucidal activity compared to other CRGs.

One of the ways to increase the bioavailability of polymers can be the production of nanoparticles (NPs). They have a unique property of combining a high charge density per unit surface and small sizes [18]. The most promising approach to obtain NPs is the utilization of green chemistry methods—to use aqua solution and biomolecules without the application of toxic solvents and synthetic cross-linking agents.

CRGs find use in the production of nanoparticles and microparticles. The presence of sulfate groups allows the formation of polyelectrolyte complexes (PECs) with oppositely charged polymers, which has proven to be very useful in the development of new drugs of various forms [19]. The advantage of PEC-based NPs is not only their high stability at different pH values, but also their increased mechanical strength compared to that of NPs obtained by other methods, such as the ionic gelation method [20]. Chitosan (CH) is most often used to form such complexes. CH is a copolymer of D-glucosamine and N-acetylglucosamine linked by β-(1→4) bonds obtained from the alkaline deacetylation of chitin, the main component of crustacean shells [21]. It exhibits low toxicity, biodegradability, good cost performance, and strong mucoadhesive properties, and it is a useful material for PEC preparation. Rodrigues et al. [22] produced complexes by mixing CH and κ-CRG, at room temperature, and they showed that the physical state of PEC formed depended on experimental conditions. Depending on the ratios, the PEC was presented as a solution or precipitate. If the CH charges are neutralized, the electrostatic repulsions are reduced or eliminated, and, in this case, the precipitation can take place. Another possibility is to use polyelectrolytes at nonstoichiometric charge ratios, as a result of which the PECs formed will remain in solution in the form of NPs [19,22].

Previously, we showed that the interaction of CRGs with the CH leads to the formation of soluble polyelectrolyte complexes (PECs) of the nanometer range [23,24]. It is known that obtaining NPs can be a way to increase the activity of the initial components. In this regard, as well as the high antiherpetic activity of κ-CRG, it was of interest to evaluate the antiviral effect of the nanosized form of PEC κ-CRG in comparison with the effect of the initial polysaccharide.

## 2. Results and Discussion

### 2.1. Characterization of the Original Polysaccharides

Chitosan was isolated from chitin of king crab shell by alkaline deacetylation. The structure of the obtained polysaccharide was confirmed by H^1^ NMR spectroscopy (Figure S1). The degree of N-deacetylation (DD) of polysaccharide was calculated based on the values of the integral intensity, which was calculated by using integrals of the peak of proton H1 of deacetylated monomer (H1) at 5.0 md and of the peak of the three protons of acetyl group (H-Ac) at 2.19 md, and it was equal to 93% [25].

Carrageenan was isolated from the red alga *Chondrus armatus* by water extraction followed by fractionation with 4% KCl solution. The structure of KCl-insoluble fraction was studied by ^13^C-NMR and IR-Fourier spectroscopy, and the obtained spectra have been compared with the spectra of polysaccharides isolated by us earlier from this specie of algae [26].

In the FTIR spectra (Figure S2), the KCl-insoluble fraction of polysaccharides from *C. armatus* showed a poorly resolved absorption band with a maximum at about 1263 and 1212 cm^−1^, indicating the presence of sulfate groups (asymmetric stretching vibrations, including two and three S=O bonds of the SO_3_ group). This spectrum showed absorption bands at 934 cm^−1^ for 3,6-anhydrogalactose (C(3′)–O–C(6′) stretching vibration) and the stretching vibration S–O bonds of 847 cm^−1^ of the axial sulfate group at C-4 of the 3-linked β-D-galactose. The two signals at 103.1 ppm and 95.9 ppm in the anomeric carbon resonance area of the ^13^C-NMR spectra (Figure S3) of KCl-insoluble polysaccharide from *C. armatus* were assigned to C-1 of the 3-linked β-D-galactose residue (G4S) and C-1 of the 4-linked 3,6-anhydro-α-D-galactose (DA) of κ-CRG. Therefore, spectroscopy data suggest that gelling polysaccharide from *C. armatus* was represented by κ-CRG. The sulfate content was 22%.

As shown earlier [12,27,28], the biological activity of polysaccharides may change significantly due to the presence of low molecular weight fractions in the sample. In this regard, the CH and κ-CRG were passed through ultrafiltration membranes to remove low molecular weight polysaccharides. Molecular weights of the polysaccharides were obtained by the viscosimetric method and were 520 ± 30 and 560 ± 45 kDa, respectively, for CH and κ-CRG.

### 2.2. Preparation and Characterization of κ-CRG-CH PEC

A number of methods are used to obtain NPs. These are ionotropic gelation, microemulsion, diffusion of emulsifying solvent, formation of PEC, and the reverse micellar method [29,30]. Of these, the method of PEC formation seems to be the most attractive. It is quite simple, not expensive, and does not require the use of organic solvents.

Soluble PECs in NPs form can be obtained in non-stoichiometric charge ratios [31]. This can be achieved, either with a significant difference in the molecular weights of the polymers, or with an excess amount of one of the components. Based on previous studies of the process of PEC formation [24], a 1:10 weight ratio (1:5 charge ratio) of CH-CRG was chosen to obtain the soluble form of PEC. The resulting complexes did not precipitate after centrifugation at 13,000 g/10 min.

#### 2.2.1. Ultracentrifugation in a Percoll Gradient

Earlier, we applied FTIR spectroscopy to prove the fact the formation of a complex between κ-CRGand CH, based on the decomposition of the spectrum into its individual components. A detailed analysis of the IR spectra is described in our previous work [32].

**Figure 1:**
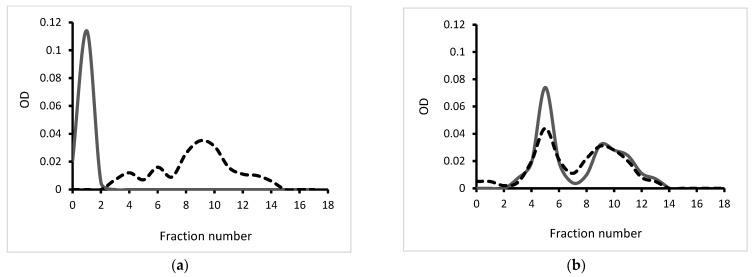
Ultracentrifugation in a Percoll gradient: (**a**) initial CH and κ-CRG and (**b**) their mixture in a ratio CH:CRG of 1:10 *w*/*w*. Contents of NH_2_-groups in CH—gray solid line; contents of SO_4_^2−^—groups in CRG—black dash line.

The use of gradient centrifugation makes it possible to separate macromolecules that differ little in size or density. This method is one of the few applications for direct registration of complex formation and has been successfully used by a number of researchers [33,34]. Here, the ultracentrifugation in a Percoll gradient was used to confirm a complex formation. The distribution curves of the initial CH and κ-CRG, as well as their mixture, are shown in Figure 1. According to the presented results, the profiles of the initial polysaccharides (Figure 1a) differ from their profiles in the mixture (Figure 1b). The coincidence of the maxima of the distribution profiles of CH and κ-CRG in the mixture proves the formation of PEC.

#### 2.2.2. Dynamic Light Scattering (DLS)

**Figure 2:**
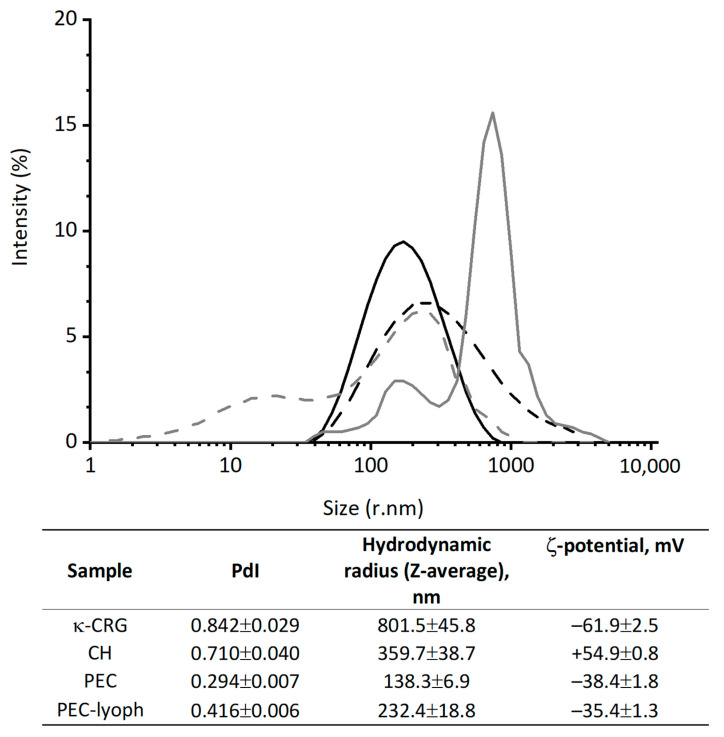
Distribution by intensity of CH-CRG 1:10 *w*/*w* particles: solid black line—freshly prepared particles; dot black line—re-dissolved particles; solid grey line—κ-CRG; dot grey line—CH.

DLS is a very powerful tool for studying the diffusion behavior of macromolecules in solution. The diffusion coefficient, and the hydrodynamic radii calculated from it, depend on the size and shape of the macromolecules. Modern data analysis, using various approaches, makes it possible to estimate the size and homogeneity of macromolecules. DLS is employed to study different types of interaction in solutions [35]. DLS data confirmed the existence of PEC of CH-CRG in these weight ratios. In the case of the mixtures of κ-CRG and CH (10:1 *w*/*w*), a significant decrease in polydispersity in comparison with initial polysaccharides and monomodal distribution of the particles were registered (Figure 2). The complex represents particles with an average hydrodynamic radius of 138 nm. A partial neutralization of κ-CRG surface charge from −61.9 mV to −38.4 mV also indicates the PEC formation. A sufficiently high negative charge of PEC indicates the electrostatic stability of the obtained particles, and this also suggests the surface localization of sulfate groups.

According to European standards, NPs are materials with a specific surface area above 60 m^2^/cm^3^ [36]. With a material density of 1, these are particles with a diameter of 100 nm. However, world practice does not adhere to such strict standards, and diameters up to 500 nm are allowed in the pharmaceutical literature [37]. Thus, the obtained PEC of κ-CRG-CH can be considered as an NP.

One of the widely used ways to store and transport NPs is lyophilization. However, they do not always retain their properties after dissolution. To control the parameters of the re-dissolved particles, freshly obtained PECs were lyophilized and, after storage for six months, they were re-dissolved, and their characteristics were determined by the DLS. As can be seen from Figure 2, the re-dissolved PEC particles have parameters quite close to those of a freshly prepared sample. Although, their polydispersity and Z-average increase slightly.

#### 2.2.3. Electron Microscopy

Scanning electron microscopy (SEM). Imaging techniques using microscopy make it possible to observe various surface characteristics of polymers, such as surface texture, shape, and thickness. Among other things, researchers prefer scanning electron microscopy (SEM) and transmission electron microscopy as reliable methods for studying the surface morphology of polymers. A comparative study of the morphology of the initial polysaccharides and their complexes can explain the difference in the macromolecular structure of the studied compounds and, as a result, their biological activity.

**Figure 3:**
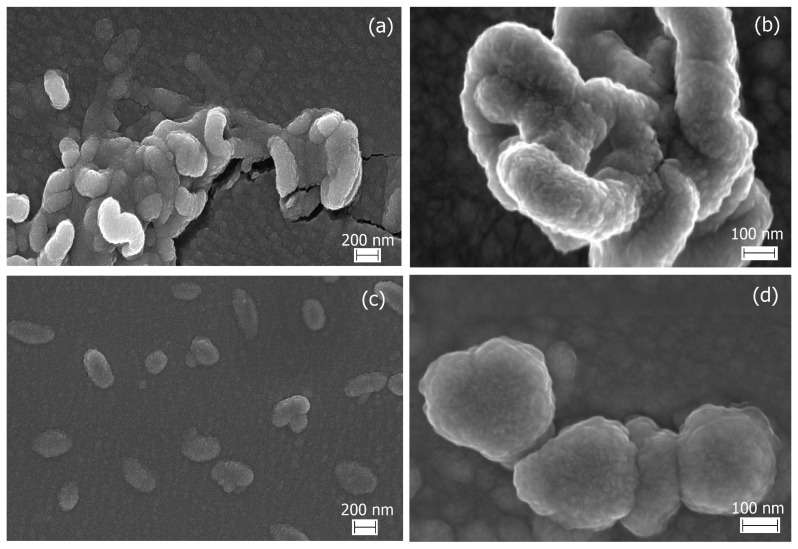
Scanning electron microscopy images of κ-CRG (**a**,**b**), C = 0.1 mg *×* mL^−1^, and PEC κ-CRG-CH 10:1 *w*/*w* (**c**,**d**).

Microstructure and surface morphology of κ-CRG and PEC were characterized by SEM. κ-CRG (Figure 3a,b) is represented by extended, curved worm-like structures of more than 1 µm, the surface of which is rough and folded. PEC (Figure 3c,d), in contrast to κ-CRG, represents compact corpuscular structures of spherical or oblong shapes of about 200–300 nm. At higher magnification (100 K), surface roughness, and wrinkling, as in the case of κ-CRG, can be seen.

Figure 3 clearly shows the changes in κ-CRG morphology after the PEC formation: polydisperse aggregated structures are transformed into more compact formations with smaller sizes. The SEM data are in good agreement with the DLS results. The size of the structures, on SEM images, is close to the particles size determined by DLS.

Transmission electron microscopy (TEM). TEM can provide more details at the atomic scale, such as crystal structure, which is more powerful and competitive than SEM.

**Figure 4:**
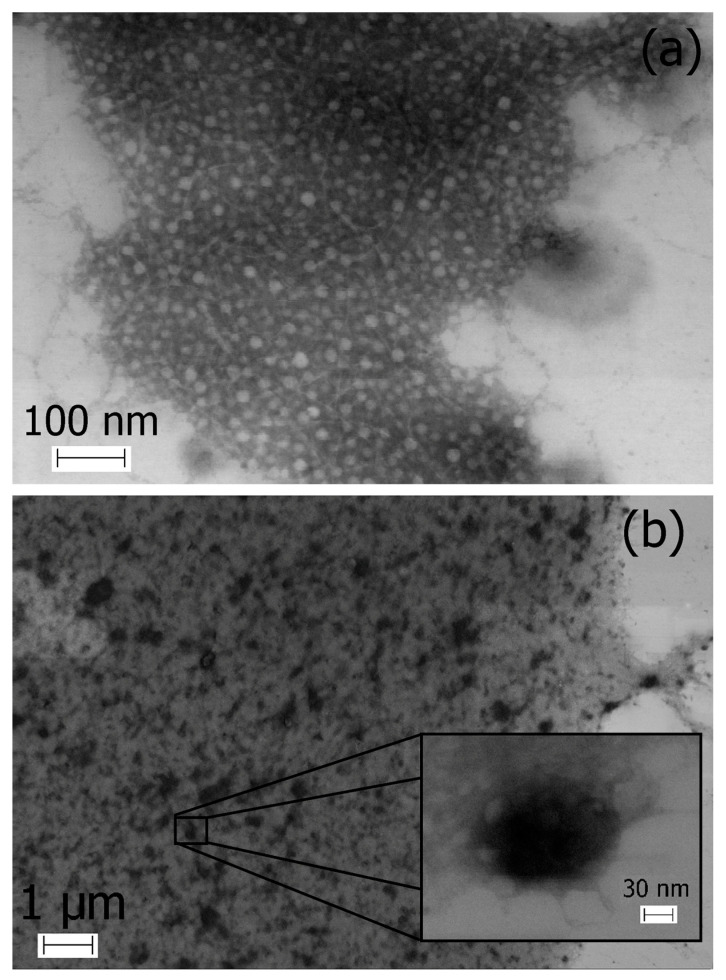
Transmission electron microscopy images of κ-CRG (**a**), C = 0.5 mg *×* mL^−1^, and PEC κ-CRG-CH 10:1 *w*/*w* (**b**).

The TEM images of κ-CRG presented in Figure 4a show the fibrous entangled structures typical of this type of polysaccharides [38,39].

TEM of the PEC (Figure 4b) revealed the presence of a large number of spherical particles of different sizes. The features of the preparation of preparations for TEM suggest the aggregation of the PEC particles during its drying on a substrate. More detailed analysis at higher magnification of single particles evidences a solid and compact structure, with an average diameter of about 150–200 nm. The PEC particle size, according to TEM data, is slightly smaller than that determined by DLS. The results were in agreement with other reports [40,41], showing that the mean size of NPs, measured by TEM, is subtle and less than that determined by DLS. This slight difference is widely considered to be influenced by the difference methods between TEM and DLS.

The centrifugation in a Percoll gradient (Figure 1) and DLS (Figure 2) indicate that κ-CRG forms stable complex with CH. The complex formation significantly reduces the polydispersity of the initial polysaccharides. According to the data of DLS and electron microscopy, the formed complex has nanometer sizes. The complex remains stable after lyophilization, followed by re-dissolution. The retention of the negative surface charge of the particles and the excess of κ-CRG in the PEC composition allows us to assume the surface localization of sulfate groups in the obtained NPs, which are known to provide antiviral activity of natural polysaccharides. Therefore, it can be supposed that the antiviral properties of CRG, which we previously established in relation to the herpes simplex virus [17], will also be characteristic of its PEC with CH.

### 2.3. Antiherpetic Activity of CH, CRG and Their PEC

Chitosan-based NPs and their biological properties are widely described [18,20,31,41,42]. NPs are used as a way to increase the antibacterial activity of the initial polysaccharide. This may be due to the small size and positive surface charge of NPs, which may improve their stability in the presence of biological cations and increase their antibacterial activity through interaction with negatively charged biological membranes and site-specific targeting in vivo [43].

CRG-based NPs are less studied. More often, this polysaccharide is used in the gel form as a matrix for incorporating different types of NPs for drug delivery or tissue engineering [44], as well as for coating for stabilization of already formed protein [45] and metal-containing NPs [46]. In this regard, it seemed interesting to us to study the antiviral properties of κ-CRG-based NPs.

The study of antiviral activity was carried out on the Vero cell line against the herpes simplex virus type 1 (HSV-1), strain L2.

The evaluation of the cytotoxicity of the polysaccharides and their PEC showed that the studied compounds had low cytotoxic activity against Vero cells. Their 50% cytotoxic concentrations (CC_50_) exceeded 2000 µg/mL (Table 1). For further study of antiviral activity, concentrations of compounds below 500 μg/mL were used.

**Figure 5:**
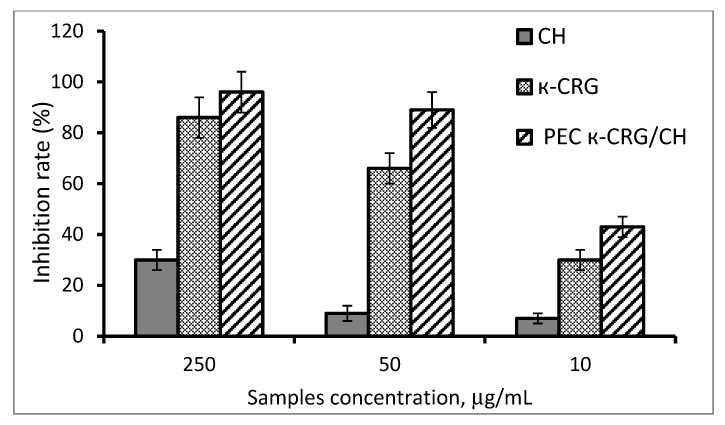
Inhibitory activity of CH, κ-CRG, and their PEC during simultaneous treatment of Vero cells with HSV-1 virus and compounds.

The antiviral effect of the studied compounds was assessed by the inhibition rate (IR, %) of the cytopathogenic effect (CPE) of the HSV-1 in Vero cells using the MTT test (Figure 5). Simultaneous treatment of Vero cells with the studied compounds and HSV-1 virus showed that CH at a concentration of 250 μg/mL inhibits the cytopathogenic effect of the virus by 35 ± 4%. At the same time, κ-CRG and PEC at this concentration almost completely suppressed the CPE of the virus: their inhibition rates did not differ significantly (*p* > 0.05) and amounted to 86 ± 9% and 96 ± 10%, respectively. However, when significantly lower concentrations of compounds (50 µg/mL) were used, PEC suppressed the CPE of the virus more effectively than κ-CRG: 89 ± 9% and 66 ± 7%, respectively (*p* ≤ 0.05) (Figure 5). At low concentrations of the tested compounds (10 μg/mL), the inhibition of viral replication using PEC was higher than that of κ-CRG—43 ± 5% and 30 ± 3%, respectively (*p* ≤ 0.05). The CH at the indicated concentrations was ineffective (Figure 5).

The low activity of CH is probably associated with a somewhat different mechanism of its antiviral activity. Although, despite these extensive published reports, the exact antiviral mechanism of chitosan and its derivatives is not fully understood [47], it is known that the CH ability to inactivate the virus by binding to its membrane was observed only at pH above 6.0 and only for certain viruses [48]. Inhibiting viral adsorption and subsequent host cell invasion was noted only for sulfated chitosan derivatives [49]. Most researchers tend to believe that the antiviral activity of CH is associated with its ability to activate immune responses. Thus, intranasal administration of CH caused activation of mucosal immune responses due to a significant increase in leukocyte infiltration and an increase in the level of pro-inflammatory cytokines in tissues [50], and it also contributed to the protection of mice from lethal doses of the influenza virus [51].

The 50% inhibitory concentrations (IC_50_) and selective indexes (SI) of the test compounds calculated by regression analysis are presented in Table 1. According to the presented data, the IC_50_ of the PEC was two times lower, and SI were two times higher than those of κ-CRG. At the same time, PEC was 13 times more effective than CH.

So, simultaneous treatment of Vero cells with the studied compounds and HSV-1 showed that the κ-CRG/CH PEC exhibits significant antiviral activity, effectively inhibiting the early stages of virus–cell interaction. Since the ratio of the initial components of κ-CRG and CH in the complex is 10:1 *w*/*w*, it is likely that the inhibitory activity of the complex is largely due to the action of κ-CRG. In turn, an increase in the selective index of PEC by two times was observed, compared to κ-CRG, and it was 13 times greated compared to CH, which indicates a greater safety and efficacy of PEC compared to its constituent components.

## 3. Materials and Methods

### 3.1. Polysacharides

A CH was obtained by alkaline treatment of crab chitin according to the published protocol [52], followed by deacetylation with the mixture of 40% aqueous solution of NaOH with isopropyl alcohol (1:16 *v*:*v*) under heating for 7 h at 100 °C. The pellet was filtered and dissolved in water acidified with hydrochloric acid (pH 3.5), dialyzed against water, and lyophilized. The DD of the CH sample was calculated according to ^1^H NMR spectroscopic data, according to the formula [25].

(1) $$\mathrm{DD}=\frac{I\left(H1\right)}{\left(I\left(H1\right)+\frac{I\left(HAc\right)}{3}\right)\;}\times 100\%$$

where *I*(*H*1) and *I*(*HAc*) represent integral intensity of the signal of *H*1 at 5.0 md and signal of protons of the acetate group at 2.19 md, respectively. The κ-CRG was isolated with hot water extraction from the red algae *Chondrus armatus* (Gigartinaceae), according to an earlier published protocol [26]. Briefly, the algae were collected at Peter the Great Bay (Sea of Japan), and they were washed with tap water in order to remove an excess of salt. Bleaching of the seaweed was achieved by maintaining the specimen in pure acetone for three days prior to being dried in the air. Dried and milled algae (50 g) were suspended in hot water (1.5 L), and the polysaccharides were extracted at 90 °C for 2 h in a water bath. The polysaccharides were fractionated into gelling KCl-insoluble and non-gelling KCl-soluble fractions. The structure of polysaccharides was established on the basis of NMR and FTIR spectroscopy data according to a previously published protocol [53]. The sulfate content was determined, as described previously [54].

### 3.2. Molecular Weight Determination

The molecular masses of polysaccharides were calculated by the Mark-Houwink-Kuhn-Sakurada equation: [η] = K × M^α^, where [η] is the intrinsic viscosity, and K and α are empirical constants for κ-CRG [55] and CH [56]. For this, the viscosity of κ-CRG and CH solutions was measured (0.1–1.0 mg/mL in 0.1 M NaCl and 2.0–10.0 mg/mL in 0.2 M NaCl/0.2 M AcOH, respectively) on a modified Ubbelohde viscometer (OKB Pushchino, Pushchino, Russia) with a capillary diameter of 0.3 mm at 25 °C, and the timing accuracy is ±0.1 s. For each concentration, five measurements were made. The intrinsic viscosity of the samples was calculated by extrapolating the ln (η) × C^−1^ dependence to infinite dilution using the least squares method, and viscosimetric molecular weights were calculated.

### 3.3. Complexes CRG:CH

The initial polysaccharides were dissolved in deionized water with stirring for 3 h and filtered through 0.45 µm. The CH-chloride solution (0.1 mg/mL) was added to equal volume of the κ-CRG solution (1 mg/mL) and stirred for 30 min at 25 °C and lyophilized.

### 3.4. Centrifugation in a Percoll Gradient

Percoll (Sigma, St. Louis, MO, USA, 26 mL, 30%) in NaCl solution (0.15 M) was placed into a 28 mL centrifuge tube. A sample of κ-CRG, CH, or their mixture (2 mL) was layered on the Percoll and centrifuged in an angled rotor Heraeus Biofuge Stratos (Hanau, Germany, Germany) at 20,000× *g* for 60 min. After centrifuging, the tube contents were removed through the top using a peristaltic pump. Fractions (1.5 mL) were collected. The density of the Percoll solution in each fraction was calculated using the refractive index determined on a RF-4 refractometer (LOMO, St. Peterburg, Russia). The presence of CH and κ-CRG in the fractions was determined by the reaction of polysaccharide amino group with 2,4,6-trinitrobenzenesulfonic acid [57] and by the reaction of sulfate group with Taylor’s blue (1,9-dimethylmethylene blue) [58], respectively. The optical density at 410 nm 535 nm, respectively, for CH and κ-CRG, was measured with a μ-Quant spectrophotometer (Bio-Tek Instruments Inc., Winuskey, VT, USA).

### 3.5. Dynamic Light Scattering (DLS) and Electrophoretic Properties of the CRG:CH Complexes

The hydrodynamic radius and ζ-potentials of the initial polysaccharides and their PEC in solution were determined using a ZetaSizer NanoZS system (Malvern, UK) operating at 633 nm. The measurements were performed at 25 °C. The parameters were automatically calculated with the instrument’s software, based on analysis of the autocorrelation function.

### 3.6. Microscopy Study

#### 3.6.1. Transmission Electron Microscopy

TEM images of samples were examined using a LIBRA-120 electron microscope (Karl Zeiss Group, Jena, Germany). The solutions of κ-CRG (0.5 mg/mL) and PEC (0.5 mg/mL κ-CRG and 0.05 mg/mL CH) in deionized water were obtained. The drops of the solutions were placed on grids coated with formvar film stabilized with carbon, desiccated using a piece of filter paper, and negatively stained for 3 min with 2% uranyl acetate. Then, the samples were carefully desiccated and examined.

#### 3.6.2. Scanning Electron Microscopy

For the scanning electron microscopy studies, solution samples in deionized water were placed onto the surface of Thermanox^®^ plastic coverslips (‘‘ThermoFisher Scientific”, Waltham, MA, USA), fixed with 2.5% glutaraldehyde, incubated for 1 h for sedimentation, and dehydrated in alcohols of increasing concentrations and acetone. Thereafter, the samples were completely dried in carbon dioxide, according to the critical-point drying method, using a BALTEC 030 (Amstetten, Germany). The samples were then placed on the surfaces of aluminium substrates and coated with chromium. The samples were analysed using a Zeiss Sigma 300 VP scanning electron microscope (Carl Zeiss Ltd., Cambridge, UK).

### 3.7. Antiherpetic Activity

#### 3.7.1. Virus and Cell Culture

In this study, HSV-1 strain L2 was obtained from N.F. Gamaleya Federal Research Centre for Epidemiology and Microbiology, Moscow, Russia. Determination of the cytotoxicity and antiviral activity of the compounds was carried out on a Vero cell culture (kidney epithelial cells of the African green monkey *Chlorocebus* sp.), obtained from N.F. Gamaleya Federal Research Centre for Epidemiology and Microbiology, Moscow, Russia. HSV-1 was grown in Vero cells using Dulbecco’s Modified Eagle’s Medium (DMEM, Biolot, St. Petersburg, Russia), which was supplemented with 10% fetal bovine serum (FBS) (Biolot, St. Petersburg, Russia) and 100 U/mL of gentamycin (Dalkhimpharm, Khabarovsk, Russia) at 37 °C in a CO_2_ incubator. In the maintenance medium, the FBS concentration decreased to 1%. The cell concentration in all experiments was 10^4^ cells/mL.

#### 3.7.2. Cytotoxicity of the Tested Compounds

The cytotoxicity evaluation of the studied samples was performed using the MTT assay, as previously described [17]. In brief, confluent Vero cells (1 × 10^4^ cells/well) in 96-well microplates were incubated with various concentrations of the tested compounds (1–2000 µg/mL) at 37 °C for 72 h (5% CO_2_); untreated cells were used as controls. MTT solution (methylthiazolyltetrazolium bromide, Sigma, St. Louis, MO, USA) was added to cells at a concentration of 5 mg/mL, following incubation for 2 h at 37 °C. Then, the MTT solution was removed, and isopropanol was added to dissolve the insoluble formazan crystals. The optical density was read at 540 nm (Labsystems Multiskan RC, Vantaa, Finland). Cytotoxicity was expressed as 50% cytotoxic concentration (CC_50_) of the tested compound, which reduced the viability of treated cells by 50%, which was compared to control cells [59]. Experiments were performed in triplicate and repeated three times.

#### 3.7.3. Anti-HSV-1 Activity of the CH, κ-CRG and Their PEC

The inhibitory effects of the CH, κ-CRG, and their PEC on HSV-1-infection in Vero cells were evaluated by the cytopathic effect (CPE) inhibition assay. Test compound concentrations ranged from 1 to 250 μg/mL; the infectious dose of the HSV-1 virus was 100 TCID50/mL (50% tissue cytopathic infectious dose). Simultaneous treatment of the cells with the tested compounds and the virus was used. The virus and samples (1:1 *v*/*v*) were applied to Vero cell monolayers, which were grown in 96-well plates, simultaneously, and incubated for one hour at 37 °C. After virus absorption, the virus–compound mixture was removed; the cells were washed, and maintenance medium was added. The plates were incubated at 37 °C for 72 h (5% CO_2_) until 90% CPE was observed in virus control compared to cell control. Antiviral activity of compounds was assessed by MTT assay, and the viral inhibition rate (IR, %) was calculated according to the formula [60]:

IR = (OD_tv_ − OD_cv_)/(OD_cd_ − OD_cv_) × 100, (2)

where OD_tv_ represents the OD of cells infected with virus and treated with the tested compounds; OD_cv_ corresponds to the OD of the untreated virus-infected cells; and, OD_cd_ is the OD of the control (untreated and noninfected) cells. The 50% inhibitory concentration (IC_50_) of each compound was determined as the compound concentration that reduced virus-mediated CPE by 50%, and it was calculated using a regression analysis of the dose–response curve [61]. The selectivity index (SI) was calculated as the ratio of CC_50_ to IC_50_ for each compound.

## 4. Conclusions

The problem of searching for compounds that can effectively act on a wide range of viral infections without harming the host organism still remains relevant. Natural sulfated polysaccharides, in this regard, seem to be very attractive. The study showed that the preparation of κ-CRG-based NPs, in the form of PEC with CH, makes it possible to increase the antiviral activity of the initial κ-CRG. This may be due to a change in the physicochemical characteristics of κ-CRG in PEC. The polysaccharide in the form of NPs has a low polydispersity, a significantly smaller size, a compact molecular shape, and, at the same time, retains a negative charge. This, in turn, can facilitate its interaction with both viral particles and the cell surface, and this thereby contributes to the manifestation of a higher antiherpetic activity.

## Figures and Tables

**Table 1:** Antiherpetic activity of CH, CRG and their PEC 1:10 *w*/*w*.

Sample	CC_50_, μg/mL	IC_50_, μg/mL	SI
κ-CRG	>2000	56 ± 8 *	36 ± 5 *
CH	>2000	374 ± 41 *	5.3 ± 0.6 *
PEC	>2000	28 ± 4	70 ± 9

* Significance of the differences between the parameters of PEC compared to other compounds (CH, κ-CRG) (*p* ≤ 0.05).

## Data Availability

The original data are available from the correspondent author on request.

## Supplementary Materials

The following supporting information can be downloaded at: https://www.mdpi.com/article/10.3390/md21040238/s1, Figure S1: ^1^H NMR spectrum of hydrochloric salt of CH; Figure S2: FTIR-spectrum of κ-CRG; Figure S3: ^13^C NMR spectrum of κ-CRG.
