# Supplementary figures and images for: Physicochemical Properties and Antiherpetic Activity of κ-Carrageenan Complex with Chitosan
Source: Mar Drugs. 2023 Apr 13;21(4):238. doi: 10.3390/md21040238 (PMC10141160; doi:10.3390/md21040238)

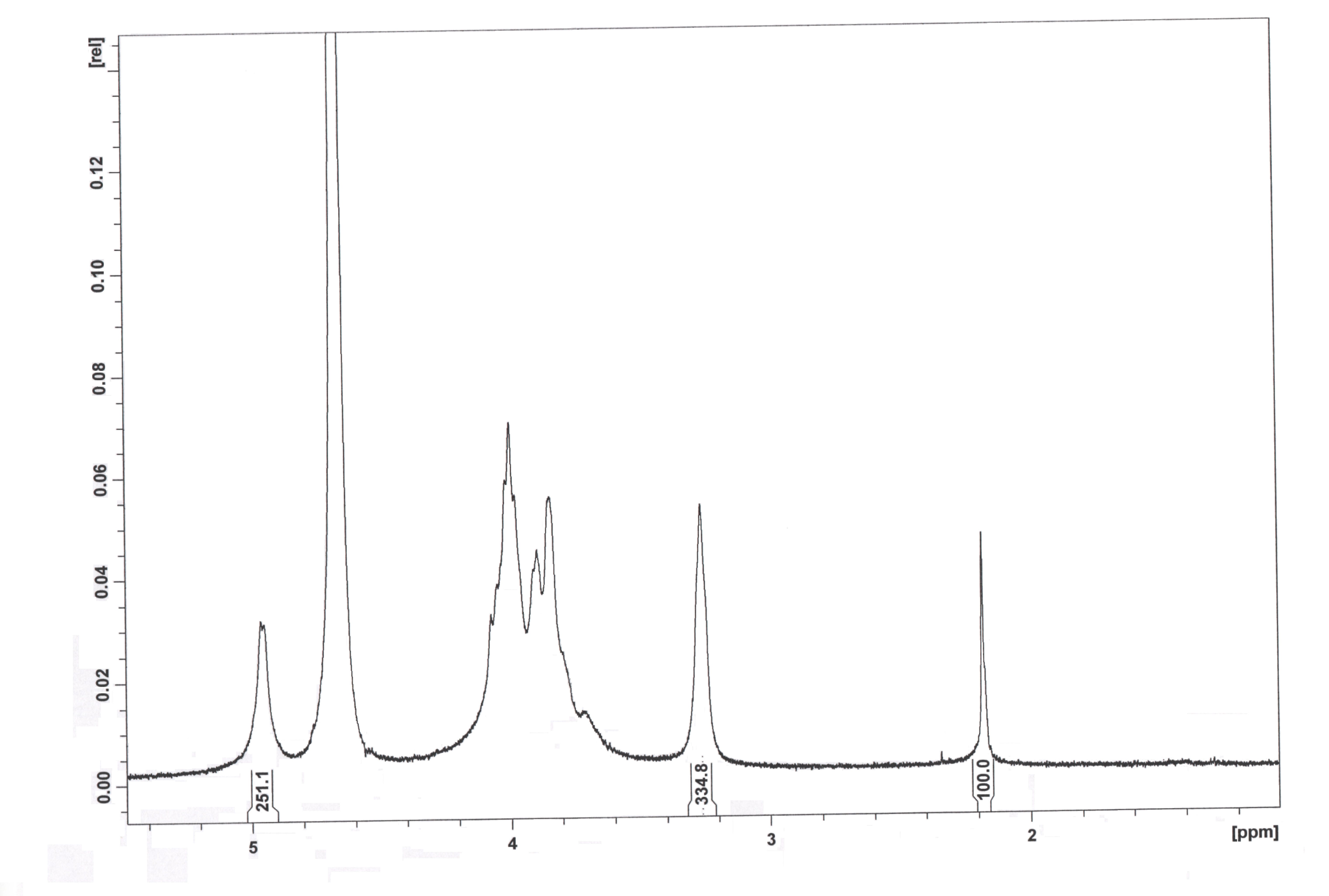

Supplement: Supplementary file 1 [file marinedrugs-21-00238-s001.zip › Figure. S1.tif]

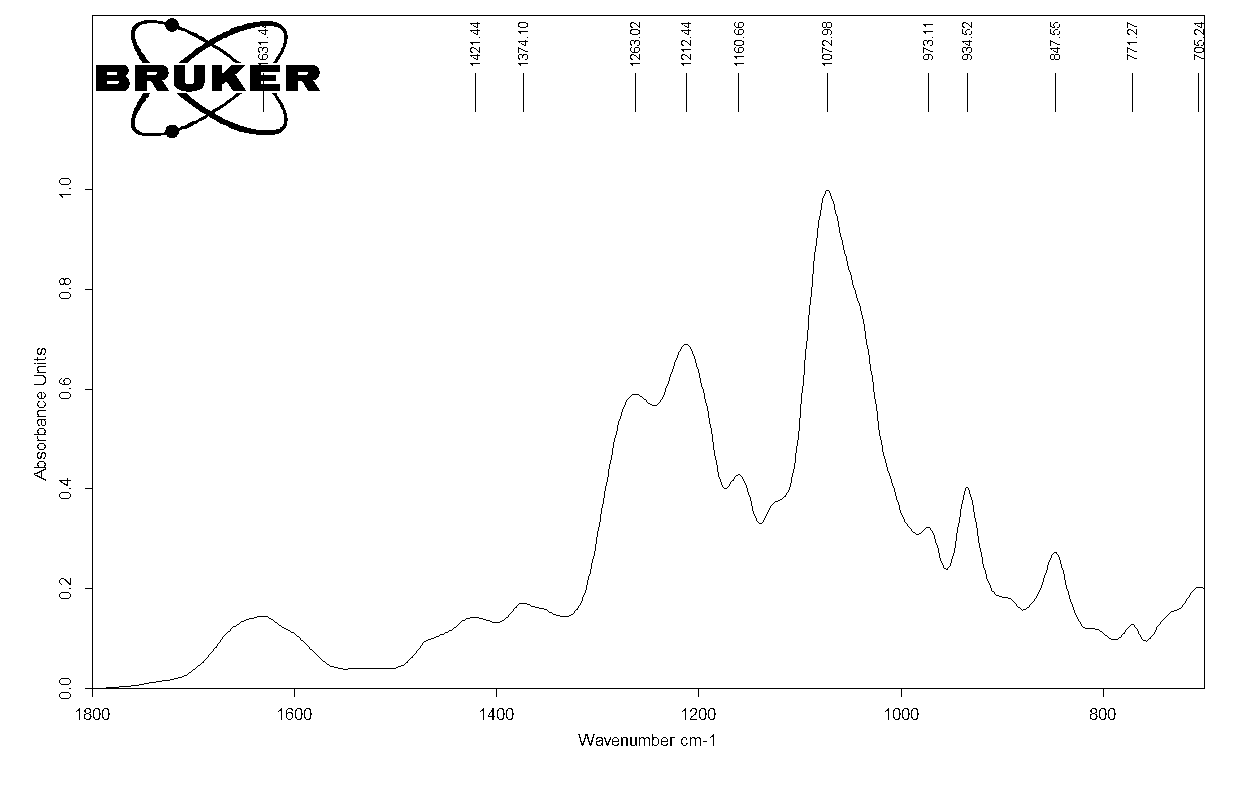

Supplement: Supplementary file 1 [file marinedrugs-21-00238-s001.zip › Figure. S2.bmp]

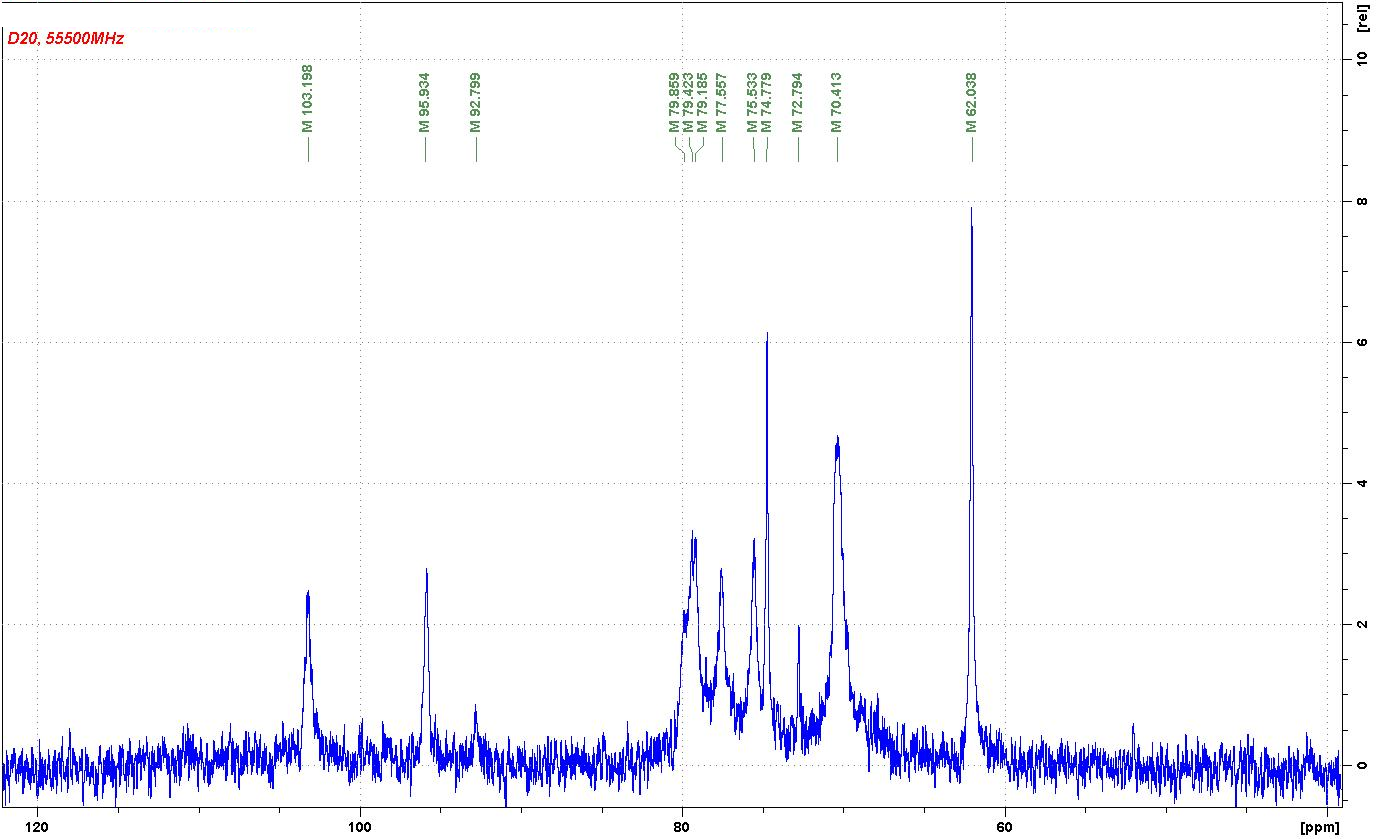

Supplement: Supplementary file 1 [file marinedrugs-21-00238-s001.zip › Figure. S3.tif]
